# Supplementary material for: Hyperspectral target detection based on graph sampling and aggregation network
Source: PLoS One. 2025 Apr 1;20(4):e0320043. doi: 10.1371/journal.pone.0320043 (PMC11960959; doi:10.1371/journal.pone.0320043)
Supplement: Attachment — Submitted filename: Response to Reviewers.docx [file pone.0320043.s003.docx]

Response to Reviewers

Dear academic editor and reviewer, hello! I have carefully reviewed my manuscript and noticed unclear explanations and insufficient descriptions. I have made revisions accordingly. The response to each viewpoint raised in the manuscript is as follows:

Editor Comments:

The opinions are as follows:

***1.Improve the analysis of the state-of-the-art by explaining the advantages and disadvantages of previous work so that you can better position the definition of the contributions of this work.***

Answer: I have reorganized the advantages of the methods mentioned in Chapter 1 and added the disadvantages of these methods. Specifically, as follows:

Detectors based on Constrained Energy Minimization (CEM) may overlook the influence of target pixels when estimating background pixels; Detectors based on adaptive consistency/cosine estimator (ACE) typically assume that there is additional noise in the background and not in the target, which often does not hold true in practical situations. In order to delve deeper into the intrinsic properties of HSI data, many researchers have been working to develop more complex modeling techniques, such as the E-CEM detection method, which requires multiple samples with feedback and the construction of multiple detectors. When the parameters are small, the algorithm performance is not stable enough; The kernel-based OSP detector maps the original space to the kernel space, enabling traditional OSP [8] methods to effectively utilize the nonlinear characteristics of HSI. However, this mapping process may result in the loss or distortion of some data information.

Xie et al. proposed a deep learning algorithm BLTSC based on background learning, which requires inputting background samples into the AAE network model for training. However, the AAE network structure is complex and requires a large amount of computational resources to learn the feature representation of the background samples during the training process. Meanwhile, BLTSC mainly focuses on background learning and lacks targeted mechanisms for target feature extraction, resulting in poor detection performance on certain datasets.

Shen et al. proposed a detection method HTD-IRN based on interpretable representation networks. Although the physical model is converted into a deep learning network to achieve compatibility between nonlinear representation and physical interpretability, there are still shortcomings in target feature extraction in complex environments, making it difficult to accurately distinguish the boundaries between the target and background when reconstructing them, resulting in detection results only on some datasets.

Zhou et al. proposed a detection algorithm CEM-VAE based on constraint energy minimization variational autoencoder, which uses background to calculate the autocorrelation matrix and introduces CEM regularization term to preserve only background information in the reconstructed samples. However, in the case of small targets or unclear target features, it cannot effectively extract target features, resulting in poor detection performance.

Tian et al. introduced the OSP concept into VAE networks and proposed an orthogonal subspace-guided variational autoencoder learning method for real background representation, OS-VAE, which can train more accurate and reliable background representation models. However, due to its emphasis on background suppression and representation, this method may to some extent neglect the learning of target features.

Li et al. proposed a hyperspectral object detection method SSROW based on spatial-spectral reconstruction and operator weighting. Although it can achieve high-accuracy detection without coarse separation of target and background samples, it requires principal component analysis (PCA) to extract feature vectors, multiple image processing, and operator construction, which increases computational complexity and operational difficulty.

In response to the above issues, the proposed hyperspectral object detection model SAGE based on graph sampling and aggregation networks has significant advantages. In terms of automatic learning feature representation, the SAGE model directly inputs all data into the model and describes the relationships between pixels by constructing adjacency matrices. In graph convolution (GNN) operation, the adjacency sparse matrix is multiplied with the node dense matrix, so that each node can fully obtain the information of its neighboring nodes. Through multiple iterations of updating node features, more representative feature representations are gradually learned. This approach differs from traditional methods and other deep learning models in that it can automatically learn complex relationships between pixels directly from image data, without the need for complex sample preprocessing, and can more accurately capture feature information of small and inconspicuous targets. Compared with other models, the SAGE model can effectively avoid the interference of irregular structures on target detection and accurately identify target pixels when processing hyperspectral data containing complex terrain and targets. In terms of improving detection accuracy and efficiency, the SAGE model reduces unnecessary computational complexity and avoids the problem of duplicate parameter settings by optimizing graph structure construction and graph convolution operations.

***2.Expand the explanation of the datasets and scientific methodology to improve understanding and repeatability.***

Answer: I have revised the analysis in the experimental results section and added examples after each analysis to improve understanding and reproducibility.

A. In terms of the dataset. I have reorganized the explanation of using the dataset, including spatial size, spatial resolution, and the number of pixels that need to be detected. I also separately added the characteristics of each dataset and the challenges it poses to the model. Specifically, as follows:

To comprehensively and effectively evaluate the performance of the method proposed in this paper, seven representative publicly available HSI datasets were selected for the experiment. The information on these datasets is shown in Table 2. These datasets have been preprocessed during download to remove noise bands such as water vapor that have less impact on noise. This not only reduces computational complexity but also optimizes data quality to a certain extent, making the experimental results more focused on the effectiveness evaluation of object detection algorithms. Due to multiple factors affecting the capture of each hyperspectral dataset, even with the same instrument, the same wavelength band can have different effects on detection. The detailed introduction is as follows:

1) Segundo dataset: captured by the Airborne Visible/Infrared Imaging Spectrometer (AVIRIS), with 224 available bands, a spatial size of 250 × 300 pixels, and a spatial resolution of 7.1m. The target pixels in this dataset are the construction area of the refinery, with a total of 2048 pixels. The characteristic of this dataset lies in its relatively large spatial size and large number of bands, which can fully test the algorithm's ability to process large-scale hyperspectral data. At the same time, as the target of the refinery construction area, its spectral characteristics have certain differences from the surrounding background, which puts high demands on the algorithm's target discrimination ability.

2) Sandiego dataset: captured by AVIRIS Naval Airport covering the San Diego area. The original dataset had 224 spectral bands, and after removing water vapor absorption and noise bands, 189 bands were retained with a spatial size of 100 × 100 pixels and a spatial resolution of approximately 3.5m. This dataset targets three aircraft. Due to the large amount of data, it was cropped into two sub-datasets for easy detection, with targets occupying 57 and 134 pixels respectively. The Sandiego dataset has a large number of spectral bands and moderate spatial resolution. Aircraft targets in complex airport backgrounds have relatively small target features and high spectral confusion with the background, which poses a serious challenge to the algorithm's ability to detect small targets in complex backgrounds.

3) ABU dataset: This dataset contains multiple subsets of data from different scenarios, all with a spatial size of 100 × 100 pixels and a spatial resolution of 7.1m (except for the abu-airport-4 subset). The abu-airport-4 dataset was obtained from hyperspectral images of the Gulfport region, with a spatial resolution of 3.4 meters and three aircraft targets. It has a total of 191 bands and 60 target pixels; The abu-urban-1 subset has 204 spectral bands and 67 residential target pixels; The abu-urban-2 subset has 207 spectral bands and 155 residential target pixels. The diversity of the ABU dataset is reflected in different target types (aircraft and residential) and the number of spectral bands, which helps to comprehensively evaluate the adaptability and effectiveness of the algorithm in various target scenarios and data features.

4) HYDICE dataset: Data from a city area in California, USA, recorded by the Hyperspectral Digital Image Acquisition Experiment (HYDICE) sensor. The image size is 80 × 100 × 162 pixels, with cars and roofs labeled, totaling 21 pixels. The characteristic of this dataset is a spatial resolution of 1m, which is relatively high and can provide more detailed ground information. However, the number of target pixels is relatively small, which requires high requirements for algorithms in small target detection and accurate positioning. At the same time, the complex ground environment in urban areas also increases the difficulty of target detection.

Figure 1 Comparison of 3D-ROC curves under various methods

B．Provide a detailed explanation of the experiment. I reorganized all the experimental analyses in Chapter 3 to make their descriptions more logical and comprehensive. I have changed the original 2D image in Figure 6 to a 3D image, which can better describe the superiority of the experimental method. Meanwhile, I modified Table 11 to include 10dB Gaussian noise in the original image data and compared the detection performance of various methods. Please refer to your opinion 3 for detailed information. Specifically, as follows:

Figure 6 shows the three-dimensional ROC curve, which represents the relationship between P_d_, P_f_, andτ. From the graph, it can be seen that the SAGE method has shown good detection performance in multiple hyperspectral datasets, achieving high true positive rates with low false positive rates and stable performance. For example, on the Segundo dataset, the ROC curve of the SAGE model is significantly better than that of other methods. In most datasets, the ROC curves of traditional CEM and ACE methods have lower True alarm rate values at low False alarm rates, indicating that these two methods are prone to producing more false positive results during detection. For other deep learning methods (SSROW, BLTSC, and HTD-IRN), these methods have shown certain advantages on individual datasets, but are not as effective as SAGE on other datasets. For example, on the Sandiego1 dataset, HTD-IRN performs similarly to SAGE, but its performance is relatively poor on the abu-airport-4 dataset.

***3.Provide a comparison in terms of noise robustness with the state-of-the-art.***

Answer：I have modified Table 11 in the original manuscript. Due to the addition of excessive noise, the original image cannot be seen clearly. Therefore, only 10dB Gaussian noise is added to the original image data to compare the detection performance of various methods. The specific analysis is as follows:

In order to analyze the robustness of the SAGE method and comparative method under noise conditions, 10dB Gaussian noise was added to the original image. From Table 11, it can be seen that the SAGE model still maintains good detection performance on most datasets, and the average accuracy of the SAGE model is much higher than that of the BLTSC and HTD-IRN models. Compared to the absence of noise, the AUC value of the method proposed in this paper has decreased, mainly due to the interference of noise between the prior target spectrum and the target to be detected after adding noise, resulting in a decrease in detection accuracy.

Table 1 AUC values of various methods under 10dB Gaussian noise.

The best values are marked in bold.

| Dataset | CEM | ACE | SSROW | BLTSC | HTD-IRN | SAGE |
| --- | --- | --- | --- | --- | --- | --- |
| Segundo | 0.7378 | 0.7585 | 0.9118 | 0.9365 | **0.9937** | 0.9911 |
| Abu-airport-4 | 0.8154 | 0.8441 | 0.9895 | **0.9978** | 0.8906 | 0.9835 |
| Sandiego1 | 0.9501 | 0.9103 | 0.9827 | 0.9910 | 0.9966 | **0.9986** |
| Sandiego2 | 0.8124 | 0.7115 | 0.9371 | 0.9637 | **0.9929** | 0.9923 |
| abu-urban-1 | 0.9779 | 0.8754 | 0.9938 | 0.9656 | 0.9841 | **0.9976** |
| abu-urban-2 | 0.8282 | 0.7991 | 0.9983 | 0.9440 | 0.9886 | **0.9989** |
| HYDICE | 0.9811 | 0.9851 | 0.9959 | 0.9449 | 0.9409 | **0.9979** |
| Average | 0.8718 | 0.8405 | 0.9727 | 0.9633 | 0.9696 | **0.9942** |

***4.Improve the explanation of the impact of the work in the conclusions***

Answer: For the conclusion section, I mainly improved the model from several aspects, including innovation and performance improvement, advantages in handling irregular structures, time efficiency and adaptability, as well as research limitations and prospects. Specifically, as follows:

The hyperspectral object detection model SAGE based on graph sampling and aggregation network proposed in this article automatically learns effective feature representations of nodes in the graph, achieves feature extraction and processing of graph data, and can fully utilize pixel relationships for feature learning. It also exhibits good robustness in complex backgrounds and noisy environments, outperforming other comparative methods. Based on the similarity between 8-neighborhood pixels and spectral cosine, a graph structure is constructed. By selecting edges with higher similarity through a threshold, the relationships between pixels are reflected through nodes and edges in the graph structure, effectively capturing irregular structures between pixels and extracting more representative features. Through extensive and in-depth experiments on seven hyperspectral image datasets, the SAGE model has demonstrated outstanding performance. Its average detection accuracy is over 99.8%, which fully demonstrates the high-precision ability of the model in hyperspectral target detection. At the same time, the SAGE model has shown excellent adaptability when dealing with different datasets. Whether it is the spatial resolution of the dataset, the difference in the number of spectral bands, or the diversity of target types and distributions, the SAGE model can flexibly respond and always maintain high detection accuracy. It also performs well in terms of time efficiency, spending less time compared to other methods and achieving efficient and accurate object detection.

Although the SAGE model has achieved good results in the field of hyperspectral object detection, there is still room for improvement. The current model has a small amount of redundant noise in the background during detection, which is a key direction for future improvement. The follow-up research plan is to further optimize the algorithm for constructing graph structures, explore more effective feature extraction and aggregation strategies, and deeply mine pixel relationships, in order to minimize the impact of background noise on detection results and continuously improve model performance.

Reviewer #1:

The comment are as follows:

***1.In Section I. INTRODUCTION, after reviewing the research status of using deep learning to construct hyperspectral object detection models, the existing problems in current research should be summarized, and a brief description should be provided on how these problems would be solved with the new proposed model.***

Answer: I have reorganized the methods of detection based on the deep learning mentioned and added their shortcomings. In the last paragraph, I briefly explained how the proposed method addresses these shortcomings. The specific changes are as follows:

In recent years, deep learning has made significant progress in fields such as pattern recognition and computer vision. Due to the better expressive ability of deep spectral features, some scholars have used deep learning to construct object detection models and achieved significant results. Xie et al. proposed a deep learning algorithm BLTSC based on background learning, which requires inputting background samples into the AAE network model for training. However, the AAE network structure is complex and requires a large amount of computational resources to learn the feature representation of the background samples during the training process. Meanwhile, BLTSC mainly focuses on background learning and lacks targeted mechanisms for target feature extraction, resulting in poor detection performance on certain datasets. Shen et al. proposed a detection method HTD-IRN based on interpretable representation networks. Although the physical model is converted into a deep learning network to achieve compatibility between nonlinear representation and physical interpretability, there are still shortcomings in target feature extraction in complex environments, making it difficult to accurately distinguish the boundaries between the target and background when reconstructing them, resulting in detection results only on some datasets. Zhou et al. proposed a detection algorithm CEM-VAE based on constraint energy minimization variational autoencoder, which uses background to calculate the autocorrelation matrix and introduces CEM regularization term to preserve only background information in the reconstructed samples. However, in the case of small targets or unclear target features, it cannot effectively extract target features, resulting in poor detection performance. Tian et al. introduced the OSP concept into VAE networks and proposed an orthogonal subspace-guided variational autoencoder learning method for real background representation, OS-VAE, which can train more accurate and reliable background representation models. However, due to its emphasis on background suppression and representation, this method may to some extent neglect the learning of target features. Li et al. proposed a hyperspectral object detection method SSROW based on spatial-spectral reconstruction and operator weighting. Although it can achieve high-accuracy detection without coarse separation of target and background samples, it requires principal component analysis (PCA) to extract feature vectors, multiple image processing, and operator construction, which increases computational complexity and operational difficulty.

In response to the above issues, the proposed hyperspectral object detection model SAGE based on graph sampling and aggregation networks has significant advantages. In terms of automatic learning feature representation, the SAGE model directly inputs all data into the model and describes the relationships between pixels by constructing adjacency matrices. In graph convolution (GNN) operation, the adjacency sparse matrix is multiplied with the node dense matrix, so that each node can fully obtain the information of its neighboring nodes. Through multiple iterations of updating node features, more representative feature representations are gradually learned. This approach differs from traditional methods and other deep learning models in that it can automatically learn complex relationships between pixels directly from image data, without the need for complex sample preprocessing, and can more accurately capture feature information of small and inconspicuous targets. Compared with other models, the SAGE model can effectively avoid the interference of irregular structures on target detection and accurately identify target pixels when processing hyperspectral data containing complex terrain and targets. In terms of improving detection accuracy and efficiency, the SAGE model reduces unnecessary computational complexity by optimizing graph structure construction and graph convolution operations.

***2.It is suggested to add a schematic diagram in part B of Section II, and conduct a more sufficient and clear description of building graph structure with the diagram.***

Answer: I have added a schematic diagram to Part B of Section 2 and included it in Figure 2, such as the "Adjacency matrix." Additionally, to better describe the process of constructing the graph structure, I have reorganized the description of this section. Specifically, as follows:

Figure 2 SAGE model flowchart.

To enable effective propagation of input data on the graph and better feature learning, it is necessary to construct a neighborhood matrix to determine the information propagation path and handle the irregularity between hyperspectral pixels. This approach does not rely on traditional assumptions of regular pixel relationships and can adapt to complex spatial and spectral feature relationships between pixels. It captures pixel relationship information in irregular structures through edges in the graph structure, enabling feature propagation and aggregation based on these relationships in subsequent operations such as graph convolution. The selection of 8 neighboring pixels is based on the consideration of pixel spatial correlation, which can better capture local structural information. The setting of the spectral cosine similarity threshold can effectively connect similar pixels and construct representative graph structures. The formula for calculating cosine similarity is:

Among them, $x_{i}$ represents the current pixel, $y_{i}$ represents neighboring pixels, $norm_{x}=\sqrt{\sum_{i} x_{i}^{2}}$，$norm_{y}=\sqrt{\sum_{i} y_{i}^{2}}$.

Specific method: Firstly, PCA is used to perform dimensionality transformation and noise filtering on the feature-enhanced results, and the total number of pixels N is obtained based on this. Next, traverse each pixel, determine its surrounding 8 neighboring pixels, calculate the coordinates of these neighboring pixels, and ensure that the neighboring pixels are within the image range and different from the current pixel. When constructing the edge index of a graph, the eight neighborhood pixels provide specific candidate neighbors. Calculate the cosine similarity between the feature vectors of each pixel and its neighboring pixels after PCA processing. If the cosine similarity is greater than the given threshold (selecting 75% of all similarity lists as the threshold), it is considered that these two pixels are similar, and the index of the neighboring pixel is added to the corresponding pixel's neighboring pixel list. Subsequently, for each neighboring pixel in the neighboring pixel list, the index pairs of the current pixel and neighboring pixels, as well as the index pairs of neighboring pixels and current pixels, are added to the edge list to construct the edges of the undirected graph. Finally, an adjacency matrix is constructed using the edge list and the total number of pixels as inputs to complete the construction of the graph structure.

**3.*Section IV of the paper should be the conclusion rather than the acknowledgement. Although the conclusion was supported by the experimental results, it did not clearly indicate the advantages of the model when dealing with irregular structures in hyperspectral data, which had been specified in Section I.***

Answer: I have changed the title of this chapter to Conclusion and reorganized the conclusions, mainly improving the innovation and performance improvement of the model, the advantages of handling irregular structures, time efficiency and adaptability, and research limitations and prospects. Specifically, as follows:

The hyperspectral object detection model SAGE based on graph sampling and aggregation network proposed in this article automatically learns effective feature representations of nodes in the graph, achieves feature extraction and processing of graph data, and can fully utilize pixel relationships for feature learning. It also exhibits good robustness in complex backgrounds and noisy environments, outperforming other comparative methods. Based on the similarity between 8-neighborhood pixels and spectral cosine, a graph structure is constructed. By selecting edges with higher similarity through a threshold, the relationships between pixels are reflected through nodes and edges in the graph structure, effectively capturing irregular structures between pixels and extracting more representative features. Through extensive and in-depth experiments on seven hyperspectral image datasets, the SAGE model has demonstrated outstanding performance. Its average detection accuracy is over 99.8%, which fully demonstrates the high-precision ability of the model in hyperspectral target detection. At the same time, the SAGE model has shown excellent adaptability when dealing with different datasets. Whether it is the spatial resolution of the dataset, the difference in the number of spectral bands, or the diversity of target types and distributions, the SAGE model can flexibly respond and always maintain high detection accuracy. It also performs well in terms of time efficiency, spending less time compared to other methods and achieving efficient and accurate object detection.

Although the SAGE model has achieved good results in the field of hyperspectral object detection, there is still room for improvement. The current model has a small amount of redundant noise in the background during detection, which is a key direction for future improvement. The follow-up research plan is to further optimize the algorithm for constructing graph structures, explore more effective feature extraction and aggregation strategies, and deeply mine pixel relationships, to minimize the impact of background noise on detection results and continuously improve model performance.

Reviewer #2:

The comment are as follows:

***1.In the related work section, although the characteristics of traditional methods (such as CEM and ACE) and deep learning methods (such as HTD-IRN) are listed, the innovative aspects of the SAGE method and its comparison with existing methods are not fully elaborated.***

Answer：

A. Describe in terms of innovation. In the revised manuscript, we provide a detailed explanation of the unique way in which the SAGE model uses graph sampling and aggregation operations to construct adjacency matrices to describe pixel relationships. In graph convolution, by multiplying the adjacency sparse matrix with the node dense matrix, each node can fully obtain the information of neighboring nodes. After multiple cycles of updating node features, more representative feature representations are gradually learned. This innovative feature learning approach is in stark contrast to traditional methods and other deep learning models, which often rely on fixed rules or assumptions to handle pixel relationships, while other deep learning models may not be able to capture complex relationships between pixels as directly and effectively in the feature learning process. For example, traditional CEM and ACE methods have relatively fixed algorithm parameters when dealing with pixel relationships, making it difficult to adapt to the complex changes in pixel relationships in hyperspectral data. On the other hand, SAGE models can automatically adapt and learn these relationships without complex sample preprocessing, and can more accurately capture the feature information of small and inconspicuous targets. We further emphasized the innovation of the SAGE model in processing irregular structures between pixels in hyperspectral data. Based on 8-neighborhood pixels and spectral cosine similarity, a graph structure is constructed, and edges with higher similarity are selected through a threshold to effectively reflect the relationships between pixels through nodes and edges in the graph structure. This method can accurately capture the irregular spatial distribution and complex spectral relationships between pixels, overcoming the limitations of traditional methods in dealing with irregular structures.

B. Detailed comparison with existing methods. In the revised manuscript, we further organized the advantages of the proposed method and supplemented its disadvantages. Compared with other models, the SAGE model can effectively avoid the interference of irregular structures on target detection and accurately identify target pixels when processing hyperspectral data containing complex terrain and targets. In terms of improving detection accuracy and efficiency, the SAGE model reduces unnecessary computational complexity and avoids the problem of duplicate parameter settings by optimizing graph structure construction and graph convolution operations.

***2. In Table II, the setting of the regularization parameter λ and the cosine similarity threshold ρ is not explained in detail. Have you considered introducing an adaptive strategy to dynamically adjust the graph structure or parameters?***

Answer: Thank you very much for raising this question. After multiple repeated experiments on this issue, I found that the regularization parameter λ had little to no effect, so I removed it in the revised manuscript. When constructing graph structures using the cosine similarity threshold ρ, we adopt a method of determining the threshold based on the cosine similarity distribution. In this method, it is necessary to first calculate the cosine similarity between all current pixels and neighboring pixels, and select 75% from this cosine similarity list as the threshold. The selection of this threshold means that 75% of the similarity values are less than or equal to this threshold, and 25% are greater than this threshold. The purpose of selecting a threshold in this way is to determine which pixels have tighter relationships (i.e. higher similarity) based on the distribution characteristics of the data when constructing the graph structure. This helps to construct a graph structure that better reflects the relationships between pixels, which is beneficial for subsequent graph convolution operations and object detection tasks.

***3. Could you provide a deeper discussion on how the model adapts to different datasets? Is the model fine-tuned for each dataset individually, or is a unified setting used across all datasets?***

Answer: This SAGE model uses a unified setting across all datasets and does not require individual fine-tuning. The specific explanation is as follows:

A. The universality of data preprocessing

In the data preprocessing stage, we adopted normalization operation (normalizing the data to intervals), which helps to make the data distribution of different datasets more regular. It can eliminate the differences in data numerical ranges caused by factors such as data collection equipment and environment, and provide more stable input for subsequent model training. For example, for datasets with different spatial resolutions and spectral band numbers (such as Segundo dataset with a spatial resolution of 7.1m and 224 bands, and HYDICE dataset with a spatial resolution of 1m and 162 bands), normalization operations make the data have similar distribution patterns before entering the model, which facilitates the model to learn the intrinsic features of the data.

B. The flexibility of graph structure

When constructing the graph structure, it is based on 8-neighborhood pixels and spectral cosine similarity, which has strong flexibility and adaptability. For different datasets, regardless of the target type, spatial resolution, and number of spectral bands, the selection of 8-neighborhood pixels can effectively capture local structural information, and spectral cosine similarity can accurately reflect the spectral similarity between pixels.

C. Adaptive learning ability of graph convolution operation

In graph convolution operations, SAGE models can gradually learn representations that adapt to different dataset features by iteratively updating node features multiple times. The multiple linear layers, batch normalization layers, and activation function (LeakyReLU) in the model work together to enable the model to automatically adjust the learning method of node features based on the specific features of the dataset. For example, on the HYDICE dataset with high spatial resolution, the model can learn the relationships between pixels more finely and capture the features of smaller targets; In ABU datasets with diverse target types (including different target types such as airplanes and residences), the model can learn the feature differences of different targets, thereby achieving accurate target detection. This adaptive learning ability allows the model to perform well on different datasets without the need for individual parameter adjustments for each dataset.

***4. In the noise disturbance experiments, the lack of comparison with other methods may not fully highlight the advantages of SAGE in this regard. Additionally, have the authors considered incorporating more evaluation metrics, rather than solely relying on AUC?***

Answer：

A. Noise interference test

Table 2 AUC values of various methods under 10dB Gaussian noise.

The best values are marked in bold.

| Dataset | CEM | ACE | SSROW | BLTSC | HTD-IRN | SAGE |
| --- | --- | --- | --- | --- | --- | --- |
| Segundo | 0.7378 | 0.7585 | 0.9118 | 0.9365 | **0.9937** | 0.9911 |
| Abu-airport-4 | 0.8154 | 0.8441 | 0.9895 | **0.9978** | 0.8906 | 0.9835 |
| Sandiego1 | 0.9501 | 0.9103 | 0.9827 | 0.9910 | 0.9966 | **0.9986** |
| Sandiego2 | 0.8124 | 0.7115 | 0.9371 | 0.9637 | **0.9929** | 0.9923 |
| abu-urban-1 | 0.9779 | 0.8754 | 0.9938 | 0.9656 | 0.9841 | **0.9976** |
| abu-urban-2 | 0.8282 | 0.7991 | 0.9983 | 0.9440 | 0.9886 | **0.9989** |
| HYDICE | 0.9811 | 0.9851 | 0.9959 | 0.9449 | 0.9409 | **0.9979** |
| Average | 0.8718 | 0.8405 | 0.9727 | 0.9633 | 0.9696 | **0.9942** |

I have modified Table 11 in the original manuscript. Due to the addition of excessive noise, the original image cannot be seen clearly. Therefore, only 10dB Gaussian noise was added to the original image data to compare the detection performance of various methods. The specific analysis is as follows: To analyze the robustness of the SAGE method and comparison method under noise conditions, 10dB Gaussian noise was added to the original image. From Table 11, it can be seen that the SAGE model still maintains good detection performance on most datasets, and the average accuracy of the SAGE model is much higher than that of the BLTSC and HTD-IRN models. Compared to the absence of noise, the AUC value of the method proposed in this paper has decreased, mainly due to the interference of noise between the prior target spectrum and the target to be detected after adding noise, resulting in a decrease in detection accuracy.

B. Evaluation indicators

For evaluation indicators, the AUC curve is crucial, and the manuscript also introduces two other comprehensive indicators for better analysis, namely AUC_OA_ and AUC_SNPR_. In addition, for better analysis, we have changed the original 2D curve in Figure 6 to a 3D curve, which represents the various relationships between P_d_, P_f_, andτ. The specific analysis is as follows:

Figure 3 Comparison of 3D-ROC curves under various methods.

From the graph, it can be seen that the SAGE method has shown good detection performance in multiple hyperspectral datasets, achieving high true positive rates with low false positive rates and stable performance. For example, on the Segundo dataset, the ROC curve of the SAGE model is significantly better than that of other methods. In most datasets, the ROC curves of traditional CEM and ACE methods have lower True alarm rate values at low False alarm rates, indicating that these two methods are prone to producing more false positive results during detection. For other deep learning methods (SSROW, BLTSC, and HTD-IRN), these methods have shown certain advantages on individual datasets, but are not as effective as SAGE on other datasets. For example, on the Sandiego1 dataset, HTD-IRN performs similarly to SAGE, but its performance is relatively poor on the abu-airport-4 dataset.

***5. The article overall seems not too bad, and I recommend a thorough re-review to improve the clarity, accuracy, and overall coherence of the paper.***

Answer: Thank you very much for your positive feedback and valuable suggestions on our paper. The main modifications made are as follows:

A. Optimize symbol representation. We carefully examined the use of all symbols in the paper and found that some letters may have different meanings, which could lead to confusion. For example, the same letter 'x' expresses different meanings, and subscripts are added to make specific distinctions.

B. Refine algorithm process description. For the description of the model method, we have organized and optimized each step description to make it clearer and easier to understand.

C. Optimize chapter structure and transitions. Re-examine the chapter structure of the paper to ensure logical coherence between each section. In the introduction section, provide a clearer explanation of the background and importance of the research question, as well as the shortcomings of existing methods and their relevance to the methodology used in this paper, so that the introduction can naturally lead to subsequent research content. Add transitional sentences and paragraphs between each chapter to help readers better understand the overall idea of the paper.

The above are my revisions to the manuscript. Please review and I hope to receive your approval. Thank you!

Date: December 21, 2024
